# Impaired visual and verbal statistical learning in children with Dyslexia in a transparent orthography

**DOI:** 10.1007/s11881-024-00321-y

**Published:** 2025-01-13

**Authors:** Angélica Mateus-Moreno, Maria Fernanda Lara-Diaz, Daniel Adrover-Roig, Eva Aguilar-Mediavilla, Gracia Jiménez-Fernández

**Affiliations:** 1 Institute for Educational Research and Innovation, Universitat de Les Illes Balears, Cta. Valldemossa, Km. 7.5 07122, Palma, Spain; 2 Department of Human Communication, Universidad Nacional de Colombia, Bogotá, Colombia; 3 Developmental and Educational Psychology Department, Universidad de Granada, Granada, Spain

**Keywords:** Statistical Learning, Reading, Transparent Orthography, Dyslexia

## Abstract

Recent research suggests that performance on Statistical Learning (SL) tasks may be lower in children with dyslexia in deep orthographies such as English. However, it is debated whether the observed difficulties may vary depending on the modality and stimulus of the task, opening a broad discussion about whether SL is a domain-general or domain-specific construct. Besides, little is known about SL in children with dyslexia who learn transparent orthographies, where the transparency of grapheme-phoneme correspondences might reduce the reliance on implicit learning processes. The present study investigates the impact of SL in Spanish, a transparent orthography, among 50 children aged 9 to 12 years, with and without dyslexia. For this purpose, we used four SL tasks to evaluate two modalities (auditory/visual) and two stimulus type (verbal/nonverbal) and evaluated both accuracy and response times on each condition. The findings reveal that children with dyslexia in Spanish exhibit lower performance on SL tasks (accuracy) compared to the control group, regardless of the modality and stimulus type used. However, children with dyslexia struggle the most with tasks that involve visual material. This indicates that children with dyslexia in transparent orthographies have particular difficulties in extracting distributional probabilistic information in the absence of explicit learning instructions. Notably, difficulties were more pronounced in visual tasks involving verbal stimuli. The present results help to better understand the underlying mechanisms involved in reading acquisition in children with dyslexia.

**Supplementary Information:**

The online version contains supplementary material available at 10.1007/s11881-024-00321-y.

## Introduction

In current reading research, there is a growing focus on viewing writing as a system characterized by statistical regularities (Sawi & Rueckl, 2019). Statistical regularities, akin to oral language, allow for the mapping of orthographic, phonological, morphological, and semantic properties within printed words. In that respect, orthographies contain regularities at many levels, and studies have shown that readers develop sensitivities to these statistical regularities through a statistical learning process (Carrillo & Alegría, 2014; Schmalz et al., 2019; Singh & Conway, 2021; Treiman & Kessler, 2022). Statistical Learning (SL) is a neurocognitive process that involves extracting patterns based on environmental statistics (Conway, 2020). Studying literacy from this perspective, as articulated by Harm and Seidenberg (2004), requires conceptualizing reading as an exercise in SL, recognizing that children, from their initial encounters with the literate language, confront numerous challenges in learning the statistical regularities of that code. This learning process demands both implicit and explicit strategies (Vandermosten et al., 2019).

The study of SL has also sparked a debate about its role in children who show difficulties in learning to read. In this sense, a phonological deficit has been described as a fundamental characteristic of dyslexia (Lyon et al., 2003; Melby-Lervåg et al., 2012; Share, 2021). Within this context, children with dyslexia exhibit deficiencies in various skills related to phonological awareness, phonological memory, and errors in speech perception (Cabbage et al., 2018; Ramus et al., 2003; Ziegler & Goswami, 2005). It is worth noting that this phonological deficit has been identified across all languages with variations in its impact depending on the type of orthography under examination.

Although phonological processing deficits are a hallmark of dyslexia (Share, 2021), they only partially account for the variability in reading difficulties observed among individuals with dyslexia. Therefore, a more general learning mechanism, such as SL, may provide further insight into the underlying causes of reading disorders (Schmalz et al., 2017). Specifically, the deficits in phonemic representation seen in this population could stem from reduced sensitivity to the statistical distribution of sounds—a core component of SL—which is crucial for reading acquisition (Ren & Wang, 2024; Vandermosten et al., 2019).

Besides, Pacton et al. (2001) showed that orthographic knowledge of graphotactic regularities can be acquired and implicitly utilized from an early stage in a child's school years. Likewise, Arciuli and Simpson (2012) observed that adults and children without reading difficulties who exhibited heightened sensitivity to statistical patterns in visual tasks, also demonstrated superior reading skills. This suggests a connection between SL and reading outcomes, specifically in English, which has a deep orthography. In that way, deep orthographies need a more advanced level of implicit learning of regularities because there are many-to-many-mappings between orthography and phonology rather than one-to-one mappings as in transparent orthographies (Arciuli, 2018). This body of research prompts interest in how this SL manifests itself in languages with transparent orthographies, such as Spanish (Nigro et al., 2016), because the variances in orthographic depth across languages could entail fundamental disparities in the use of SL when learning to read.

To date, research on SL deficits in children with dyslexia in transparent orthographies remains limited and contradictory. Reviews, such as the one by Singh and Conway (2021), include studies conducted in transparent orthographies like Dutch and Swedish, which report mixed results regarding SL performance. For instance, Hedenius et al. (2021) assessed implicit learning in Swedish children with dyslexia using a serial reaction time task and found that learning consolidation was more impaired in the group with dyslexia than in controls, even after a prolonged initial learning session.

However, Staels and Broeck (2015) found no evidence of an SL deficit in Dutch children and adolescents with dyslexia when using the Hebb repetition learning task. In a follow-up study, Staels and Broeck (2017) reported similar implicit learning performance between children with dyslexia and controls using a serial reaction time task. Similarly, van der Kleij et al. (2018) found that while Dutch children with dyslexia had longer reaction times, they did not differ in how well or quickly they learned an implicit sequential and spatial learning task. Further complicating the picture, Van Witteloostuijn et al. (2019, 2021) found no difference in SL performance between Dutch children with dyslexia and controls and highlighted that the clinical relevance of SL for dyslexia may be limited. In contrast, Vandermosten et al. (2019) showed that Dutch readers with dyslexia make less use of statistical cues in oral language, leading to less differentiated phonemic categories and an increased risk of impaired phoneme-grapheme correspondence.

This body of mixed findings suggests that the evidence for SL deficits in children with dyslexia, particularly in transparent orthographies, is not conclusive. Nevertheless, the meta-analysis by Lee et al. (2022) points to a general disadvantage in SL for individuals with dyslexia across both transparent and opaque orthographies. This disparity underscores the need for further investigation of the relationship between SL and dyslexia in transparent orthographies.

Our study seeks to contribute to this ongoing debate by examining SL in the context of Spanish, a less explored yet highly transparent orthography. Spanish, with its regular and predictable correspondence between letters and sounds, stands out as the most transparent orthography compared to Dutch and Swedish, which have more variable orthography-phonology rules. This level of transparency in Spanish offers a unique opportunity to better understand how SL skills influence children with dyslexia (Dobó et al., 2021; Von Koss Torkildsen et al., 2019). This clearer context may help disentangle the specific contributions of SL to reading acquisition in dyslexia, particularly in transparent orthographies where phoneme-grapheme correspondences are generally more straightforward, yet some phonemes still present challenges for children with dyslexia (Nigro et al., 2016), offering new insights into the potential role of SL in dyslexia within this linguistic framework.

Moreover, different studies have observed that SL task performance may vary depending on the modality and/or stimulus type used (we use modality to refer to the sensory input of the stimulus: visual/auditory and stimulus type to denote the verbal-nonverbal distinction in our tasks), showing how these input characteristics influence SL performance. This has generated an ongoing debate as to whether SL should be categorized as a domain-general construct by which cognitive systems discover underlying distributional properties independent of sensory input or a domain-specific mechanism that is subject to modality and stimulus type-specific constraints (Frost et al., 2015). Regardless of this debate, it has been proposed that SL may indeed exhibit domain specificity, influenced by factors such as modality and stimulus type, with this domain specificity having an impact on processes related to written literacy (Sawi & Rueckl, 2019; Siegelman & Frost, 2015).

Nonetheless, there are conflicting results on the involvement of task modality and stimulus type in people with dyslexia (Frost et al., 2015; Lum et al., 2013; Gabay et al., 2015). For example, Arciuli and Simpson (2012) found a direct association between visual SL and reading ability in the general population. However, these results are contradictory to those from Ozernov-Palchik et al. (2023) who found differences only in auditory modality SL tasks for adults with dyslexia, who have impairment in auditory statistical learning that is associated with reading difficulty. In the same vein, Vandermosten et al. (2019) reported that readers with dyslexia exhibited poorer outcomes on auditory tasks and concluded that this group makes minor use of the statistical cues embedded in spoken language, resulting in less distinct phonemic categories and, therefore, a higher risk of not being able to establish sound phonemic categories. In contrast, Kligler and Gabay (2023) found that in young adults SL performance was worse in the dyslexia group than among typical readers in both visual and auditory modalities, describing that the SL performance observed in dyslexia stems from a domain-general impairment and that cross-modal information can be recruited to support the role of SL in dyslexia.

In children with dyslexia, there are also contradictory results between modality and stimulus type, showing a low consensus on the role of these variables in the performance of children with dyslexia in SL tasks (for a review: van Witteloostuijn et al., 2019; Singh & Conway, 2021; Schmalz et al., 2019; Ballan et al., 2023). The differences among these results suggest the need to investigate SL in dyslexia using a paradigm that combines similar tasks across different sensory modalities and includes verbal and nonverbal stimuli. In this respect, Elleman et al. (2019) highlight the importance of exploring domain-specific SL across various presentation modalities in explaining the reading and spelling challenges experienced by children with dyslexia.

Therefore, the present study aims to explore SL skills in children with dyslexia learning to read in a transparent language (Spanish) compared with those of typical reading children; and to assess if there is a difference in performance in SL tasks concerning the modality or stimulus used. For this purpose, this study used four SL tasks to evaluate two modalities (auditory/visual) and two stimulus type (verbal/nonverbal). We hypothesize that the children with dyslexia learning to read in Spanish have a lower performance in SL tasks compared with the control group, although Spanish is a transparent orthography. Specifically, we expect that children with dyslexia will have a lower performance in auditory modality SL tasks, due to the fact that phonological deficit described in this population, and about the stimulus type we expect that regardless of the modality, children with dyslexia will have a reduced performance in verbal tasks, which involve a greater linguistic skill and may require a more complex challenge and a task closer to the reading tasks they perform in everyday life.

## Method

### Participants

The sample consisted of 50 Colombian children between 9 and 12 years of age, who were divided into two groups: the study group with a diagnosis of dyslexia (DD group), and the control group composed of typically developing children (TD group). All participants were enrolled in school in the grade corresponding to their age, and each group (DD group and TD group) included twenty-five participants (9 girls in each group, accounting for 36% of the sample).

The inclusion criteria for the DD group were, a) diagnosis of dyslexia not exceeding two years; b) referral by teachers or speech and language therapists due to reading difficulties; c) IQ within the age-appropriate average range (mean = 100; SD = 15); and d) no neurological, cognitive, or sensory issues. Conversely, TD group criteria were, a) no history of language or reading problems; b) no neurological, cognitive, or sensory issues; and c) IQ within the age-appropriate average range (Mean = 100 *SD* = 15).

Accordingly, all participants in the dyslexia group had a previous diagnosis of dyslexia and were referred by psychologists and therapists at their schools. To confirm the inclusion criteria several tests were administered to participants. Nonverbal intelligence was assessed using the *Matrices subtest* of the Kaufman Brief Intelligence Test (K-BIT; Kaufman & Kaufman, 1997), which measures the ability to solve reasoning problems with the assistance of visual stimuli. To confirm the presence of a reading disorder or discard it, the pseudoword decoding and word reading sub-tests of the *PROLEC- R test* (Cuetos et al., 2007) were administered. Table 1 presents the sociodemographic data and average scores of non-verbal intelligence and reading measures for both groups.

**Table 1:** Characterization of DD group and TD group

	DD Mean (*SD*)	TD Mean (*SD*)	*U*	*p*
*n*	25	25		
Age (years)	10.59 (1.16)	10.57 (1.18)	312.5	1
Non-verbal IQ	93.20 (3.25)	93.28 (8.88)	298.5	0.785
WR (a)	23.6 (10.9)	39.2 (1.0)	10.5	** < 0.001**
WR (s)	181.6 (135.5)	41.12 (13.6)	14.5	** < 0.001**
PR (a)	19.0 (10.5)	38.2 (2.0)	2.0	** < 0.001**
PR (s)	190.12 (116.2)	63.9 (15.8)	29.0	** < 0.001**

WR: Word reading Task (number of words read); PR: Pseudowords Reading Task; (a): accuracy, (s) time seconds; *SD*: Standard Deviation; IQ: Intelligence quotient

### Experimental tasks

Four experimental tasks were developed to assess SL of both verbal and nonverbal regularities in both the visual and auditory modalities. The verbal tasks followed the principles of the Artificial Grammatical Learning paradigm (AGL; Reber, 1967). In this paradigm, participants are exposed to sequences generated from an artificial grammar that dictates the order in which particular stimuli can occur in the sequence (Singh & Conway, 2021); after a familiarization phase, participants engage in a forced-choice phase, where they make grammaticality judgments on novel sequences governed by the same grammatical rules as in the previous phase. The non-verbal tasks followed the principles of triplet task (Saffran et al., 1996). In this paradigm, participants are exposed to stimuli that conform to three pictures (see Arciuli & Simpson, 2012) in the familiarization phase, after participants are given a two-alternative forced-choice phase judging which of two triplets is “familiar” (Singh & Conway, 2021).

In the familiarization phase, the instruction given to the participants was: *"Now you are going to see some characters that look very strange, look at them carefully, and if you find a repeated character, press the key"*. The instruction for all four tasks included a coverage task designed to ensure that participants attended to the stimuli displayed on the screen. For the auditory tasks, the instructions were similar: participants were required to identify auditory stimuli that were repeated within the presented string by pressing a key each time they detected two identical sounds. Data from participants who failed to identify at least 50% of the repeated stimuli in the coverage task were excluded from the analysis. Descriptive analyses of the coverage task are provided in the supplementary material.

In the forced-choice phase, the instruction given by the evaluator was: *"Now you are going to see two stimuli, one of them follows the rules of the ones you have seen before. Choose by pressing the key the one that seems to follow the rule and is more familiar to you”*. In this phase, participants had to select between two alternative stimuli. This type of methodology has been used in previous studies, demonstrating that children and adults show superior learning to chance in this explicit decision phase (Arciuli & Simpson, 2011; Arciuli and Simpson, 2012; Tong et al., 2019; Von Koss Torkildsen et al.,; 2019). Similarly, Qi et al. (2019) have also reported good psychometric properties for assessing SL in children and adults (Cronbach's alpha > 7).

All the SL tasks designed were previously tested with young adults and children of similar ages to the study groups. This initial testing allowed validation of the instructions, response format, and stimuli used. To identify whether performance in the forced-choice phase was greater than chance as a two-alternative choice task (accuracy 50%), a *t*-test was performed, and cases where learning was less than 50% were discarded.

The four SL tasks designed are described below.

### *Visual non-verbal (VNV) SL task* (Fig. 1a)

**Fig. 1:**
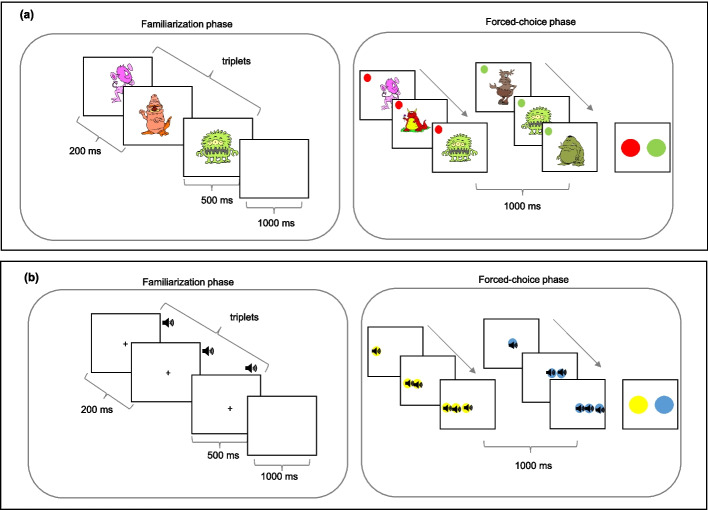
Representation of the visual and auditory non-verbal SL tasks. Note. Schematic representation of the Non-verbal SL tasks. (**a**) Visual Non-verbal SL Task; (**b**) Auditory Non-verbal SL Task. In the forced-choice phase, the triplets were shown to maintain the same presentation intervals as in the familiarization phase including a symbol of a representative color (circle) that allows the participant to identify the stimuli corresponding to each of the triplets and make a choice as to which of the triplets was more familiar

This task used 18 artificial figures that were unknown to the participants and contained no semantic content with real scenarios. These figures were obtained from https://www.clipart.com, with four figures employed for the task trials and 12 were used to create four triplets following probabilistic presentation principles from triplet task based on the task used by Arciuli and Simpson (2012). During the familiarization phase, the stimuli were presented individually in the center of the screen. Each stimulus lasted 500 ms, with 200 ms as the inter-stimulus interval (ISI). To differentiate each triplet (trial), a 1000 ms interval was used as the inter-trial interval (ITI).

The familiarization phase consisted of 24 random repetitions of each of the four created triplets, maintaining the order established by the stimulus within each triplet. Twelve stimuli were chosen for the experiment, and these were divided into four groups of three (four triplets [trials]: ABC, DEF, GHI, JKL).

During the forced-choice phase, four new groups of triplets were formed, which were different from those presented in the familiarization phase and not following the rules used in this phase (ABC, DEF, GHI, JKL). These new triplets had not been presented in the familiarization phase and were impossible triplets. Each triplet that followed the rules used in the familiarization phase was presented aside from each impossible triplet on four separate occasions with the order of presentation counterbalanced. For this phase, a color was assigned to each of the triplets, facilitating their identification and allowing their subsequent selection. In this case, the triplets were presented following the same methodology as in the familiarization phase. At the end of the presentation of the two triplets (possible/impossible), a blank screen appeared with the colors representing each triplet. At this point, the triplet that was most familiar to him/her had to be chosen.

### *Auditory non-verbal (ANV) SL task* (Fig. 1b).

An inventory of 18 non-verbal sounds (from freely available environmental sources: https://pixabay.com/) was compiled and organized into triplets. The acoustic characteristics of all stimuli were adjusted using the Audacity program to ensure uniform duration and intensity for all stimuli. Of these sounds, four were used for task trials, and 12 were used to create triplets. Four triplets were organized following the experimental structure of the previous task: ABC, DEF, GHI, and JKL.

The familiarization phase consisted of 24 random repetitions of the four created triplets for a total of 96 presentations of the auditory stimuli, maintaining the order established by the stimuli within each triplet. During the familiarization phase, sounds were presented one at a time, with the screen blank with a fixation point in the center of the screen. The duration of intra- and inter-triplet stimuli was consistent across all experimental tasks as previously described for the visual SL task. For the forced-choice phase, four triplets that followed the rules presented in the familiarization phase were created and presented together with triplets that did not follow these rules (impossible triplets). Participants listened to both triplets and then selected the triplet that was most familiar to them.

### *Visual verbal (VV) SL task* (Fig. 2a)

**Fig. 2:**
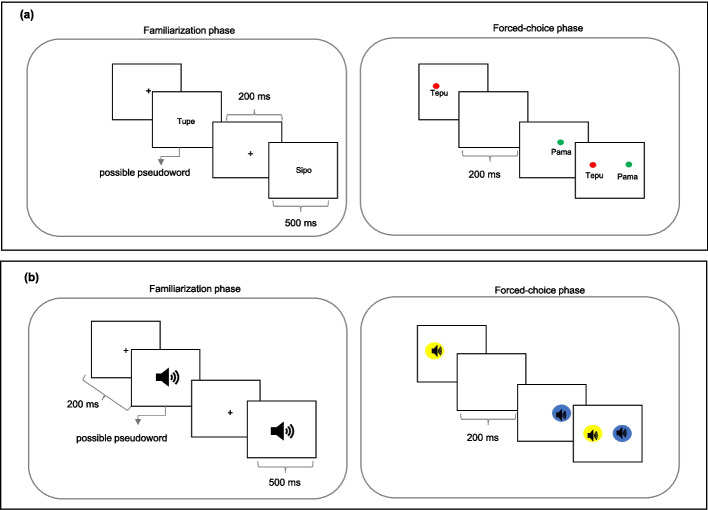
Representation of the visual and auditory verbal SL tasks. Note. Schematic representation of the verbal SL tasks administered in the present study. (**a**) Visual verbal SL task; (**b**) Auditory verbal SL task. In the forced-choice phase, the stimuli maintain the same presentation intervals as in the familiarization phase, including a symbol of a representative color (circle) that allows the participant to identify the stimuli corresponding and make a choice as to which of the verbal stimuli was more familiar

Participants observed a series of two-syllables CVCV pseudowords based on predetermined probabilistic rules using the letters: M, S, T, P, L, and F. These six consonants were organized according to artificial graphotactic rules absent in the Spanish language, which restricted their allowable positions [position 1 (C1) or position (C2) within the pseudoword CVCV]. In the first consonant position (C1) only the following letters could appear: M, S, and T; while in the second consonant position (C2) only the consonants P, L, and F could appear.

During the familiarization phase, words created using these graphotactic rules were sequentially presented on a white background. The familiarization stream featured 96 presentations of the 12 words (possible pseudowords). Repetitions were randomly presented among participants. Each stimulus lasted 500 ms, with an inter-stimulus interval of 200 ms. For the forced-choice phase, 16 stimuli (possible pseudowords) were created that followed the same graphotactic position rules of C1 and C2 of the familiarization phase, and 16 stimuli that did not (impossible pseudowords). The 16 impossible pseudowords were created by interchanging the position of the consonant, so that C1 consisted of the letters: P, L, and F, and C2 consisted of the letters: M, S, and T. Both the familiarization phase and the forced-choice phase were composed of three trials, before starting with the evaluation stimuli. Each possible pseudoword that followed the graphotactic position rules was presented with each impossible pseudoword with the order of presentation counterbalanced and the participant selected the word he/she thought was correct.

### *Auditory verbal (AV) SL task* (Fig. 2b).

Participants were presented with a series of disyllabic CVCV pseudowords following predefined probabilistic rules following the same methodology of the VV task utilizing six consonants organized by artificial graphotactic rules. In the first consonant position (C1) only the following letters could appear B, D, N while in the second consonant position (C2) only the consonants could appear: G, K, J. Words were recorded and presented with consistent intensity, frequency, and duration. The number of repetitions of the possible stimuli in the familiarization and forced-choice phase was the same for all SL tasks. In the forced-choice phase, 16 stimuli (possible pseudowords) were created that followed the same graphotactic position rules of C1 and C2 of the familiarization phase, and 16 stimuli that did not (impossible pseudowords). Participants judged which of two words appeared more familiar (possible pseudoword / impossible pseudoword), following the same methodology as the previous SL tasks.

### Procedure

This study received approval from the Research Ethics Committee of the Faculty of Medicine at the National University of Colombia. All participants and their parents signed a written informed consent at the beginning of the study. SL tasks were developed and subjected to pilot testing with both adults and children. This phase served to refine instructions, establish optimal stimulus presentation durations, and ensure participants' comprehension of response button usage. Data collection occurred across two sessions, each lasting approximately 45 to 60 min. In the first session, the nonverbal intelligence test and two SL tasks were carried out by participants. In the second session, the two remaining SL tasks and the reading test (*PROLEC-R*) were completed. Each SL task was alternated with the other measures taken within this study and the participant was given a break to continue with the next SL task. The interval between the first and second data collection sessions was one week.

The experimental tasks were administered using *E-prime* *3.0* software, with the order of task presentation being counterbalanced across participants. A 23-inch monitor, positioned on a tabletop desk at a fixed distance of 60 cm from each participant seated in a stationary chair, was employed for all tasks.

### Statistical analysis

Descriptive statistics were obtained for raw scores of the intelligence and reading tests. For the SL tasks, we analyzed both Response Times (RT) and Accuracy (A) using the statistical software *SPSS* version 22.0.

To ascertain that the performance of the SL tasks occurred above chance (accuracy 50%), a one-sample *t*-test was used. Outliers were identified using the median plus or minus two standard deviation criteria. We discarded all cases in which the RT and Accuracy were 2 SDs above or below the group's mean. This constituted 4% of the data.

To compare the general performance of DD and TD groups in the four SL tasks, a 2 × 2 × 2 mixed analysis of variance (ANOVA) was conducted with Group (DD, TD) as a between-subjects factor, and modality task (visual, auditory) and stimulus type (verbal, nonverbal) as within-subjects factors on both Accuracy and RTs. A significance level of *p* < 0.05 was used for all comparisons. For all tests of simple effects involving post hoc multiple comparisons, the Bonferroni correction was applied (*SPSS*’s *α*-corrected values of *p* are reported). *Greenhouse–Geisser* (G-G) correction was applied to correct the degrees of freedom in those cases in which the sphericity assumption of the repeated measures designs was violated.

## Results

Data from the coverage task performed during the familiarization phase were analyzed to ensure participants' engagement with the stimuli. All participants were required to have a mean score *> 0.5* to confirm adequate exposure during this phase. The mean scores for the coverage task across the entire sample for each condition were as follows: visual-verbal task = 0.884, visual nonverbal task = 0.598, auditory verbal task = 0.629, and auditory nonverbal task = 0.644. A detailed analysis of this task is available in the supplementary material.

To assess whether SL occurred regardless of group, one-sample *t*-tests were conducted to compare each group's performance against the chance probability level (50%) of incidental learning for each SL task. Altogether, participants demonstrated SL with average accuracy scores significantly above chance for each task: visual non-verbal: 61.2% (*t* (49) = 4.2, *p* < 0.0001), visual verbal: 55% (*t* (49) = 1.9, *p* = 0.04), auditory non-verbal: 60% (*t* (49) = 5.0, *p* < 0.0001), and auditory verbal: 70% (*t* (49) = 8.7, *p* < 0.0001). Descriptive statistics for each group in the fourth SL task can be consulted in Table 2.

**Table 2:** Mean and standard deviations for the four SL tasks for each group

Modality	Type	Measures	DD Mean (*SD)*	TD Mean (*SD*)
Visual	NV	Accuracy	0.47 (0.082)	0.76 (0.155)
		RT (ms)	1071.6 (559.8)	798.9 (546.5)
	V	Accuracy	0.43 (0.110)	0.67 (0.125)
		RT (ms)	1329.3 (535.7)	1020.2 (400.9)
Auditory	NV	Accuracy	0.55 (0.112)	0.66 (0.155)
		RT (ms)	759.5 (318.1)	831.7 (499.0)
	V	Accuracy	0.58 (0.120)	0.81 (0.115)
		RT (ms)	771.9 (339.7)	649.5 (218.7)

NV: Non-verbal; V: Verbal; RT: Response Time; *SD*: Standard Deviation. Reaction times are provided in milliseconds and accuracies represent the rate between correct answers and the total number of trials.

### Accuracy: Effects of group, modality and stimulus type

The 2 × 2 × 2 mixed ANOVA conducted with Group (DD, TD) as the between-subjects factor, and the modality of task (visual, auditory) and stimulus type (verbal, nonverbal) as within-subject factors showed a main effect of Group, [*F*(1, 47) = 161.3, *p* < 0.001, ${\upeta }_{ p}^{2}$ = 0.77], revealing TD were more accurate than DD in SL tasks regardless of modality or stimulus type. There were also main effects of Modality [*F*(1,47) = 20.934, *p* < 0.001, ${\upeta }_{ p}^{2}$ = 0.30], but not for Stimulus type [*F*(1, 47) = 0.373, *p* = 0.544, ${\upeta }_{ p}^{2}$ = 0.008], with higher accuracy for the auditory SL task relative to the visual SL task.

Results also revealed significant interactions of Modality x Stimulus type, [*F*(1,47) = 17.275, *p* < 0.001, ${\upeta }_{ p}^{2}$ = 0.26], Group x Modality [*F*(1,47) = 9.926, *p* = 003, ${\upeta }_{ p}^{2}$ = 0.17] and Group x Modality x Stimulus type [*F*(1,47) = 4.525, *p* = 0.039, ${\upeta }_{ p}^{2}$ = 0.08].

**Fig. 3:**
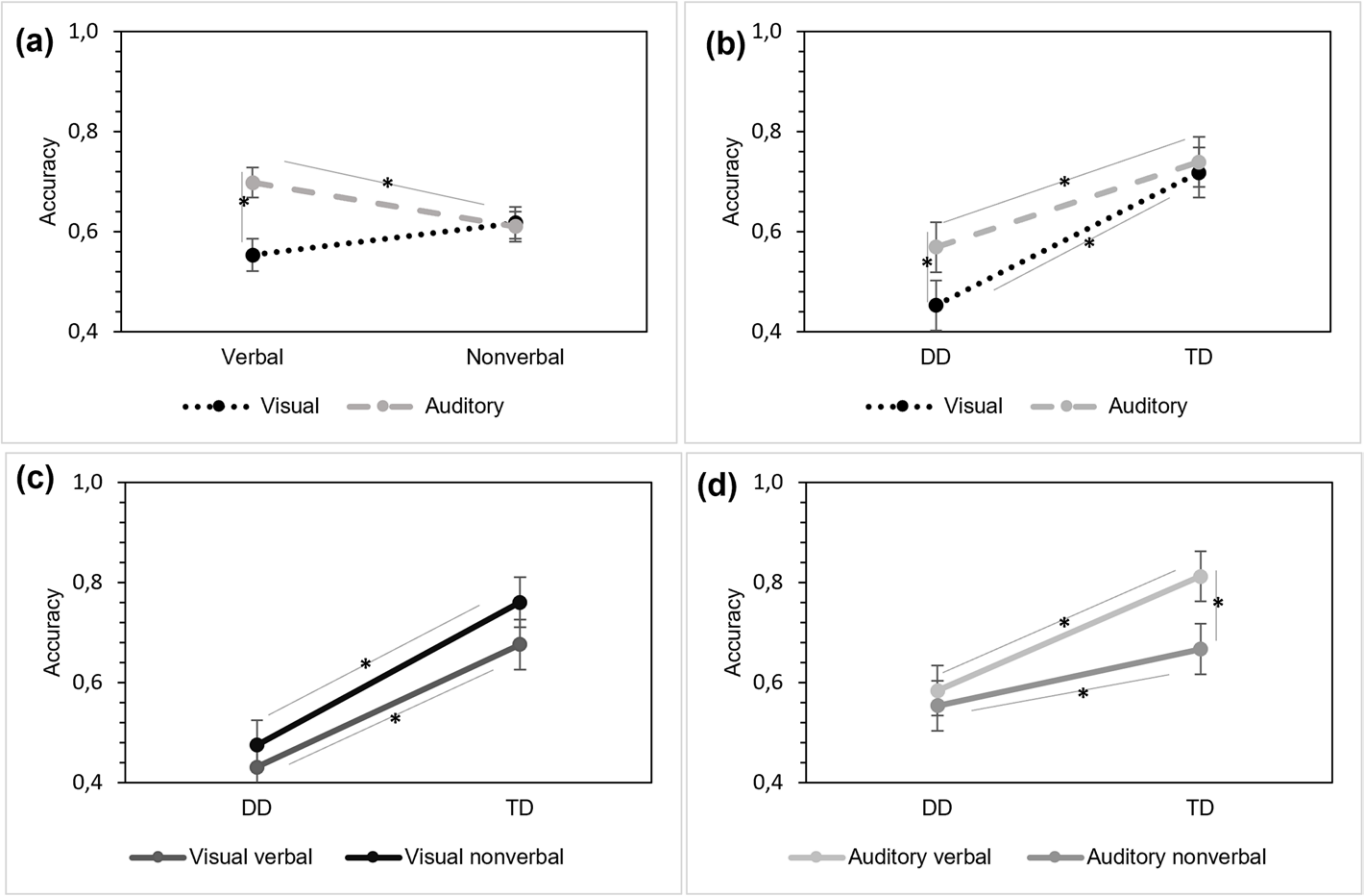
Accuracy for the four SL tasks. Note: Accuracy as measured by the percentage of correct responses: (**a**) for each modality (visual/auditory) by stimulus type (verbal/nonverbal); (**b**) for each modality broken by group (**c**), and (**d**) for each SL task broken by group. Error bars display the standard error of the mean; *p < 0.05. The line (/) shows the interaction between variables (modality, stimulus type, and group). DD: dyslexia group, TD: Control group

Post hoc comparisons revealed that performance is significantly lower for visual tasks with verbal stimuli compared to auditory tasks with the same stimuli (*MD* = −0.144, p_bonf_ < 0.001); however, task performance is equivalent for the visual and the auditory SL task with non-verbal stimuli (*MD* = 0.007, p_bonf_ = 1). In the intramodality analysis, verbal and non-verbal stimuli do not exhibit an effect of stimulus type in the visual modality (*MD* = −0.064, p_bonf_ = 0.129); however, in the auditory modality this effect is observed (*M*D = 0.087, p_bonf_ = 0.011; see Fig. 3a).

For the Group x Modality interaction, the post hoc comparisons showed that the DD group had lower accuracy in the visual SL tasks compared to the TD group (*MD* = −0.265, p_bonf_ = < 0.001). For the DD group, auditory SL obtained higher accuracy values than visual SL (*MD* = −0.116, p_bonf_ = < 0.001), which did not differ in the TD group (*MD* = −0.021, p_bonf_ = 1.000) (see Fig. 3b).

The Group x Modality x Stimulus Type interaction revealed that the TD group achieved higher accuracy scores in the auditory-verbal task compared to the auditory non-verbal SL task (*MD* = 0.145, p_bonf_ = 0.007), a difference not observed in the DD group (*MD* = 0.030, p_bonf_ = 1.000, see Figs. 3c and 3d). Overall, the TD group outperformed the DD group in both visual (*MD* = −0.265, p_bonf_ = < 0.001), and auditory SL tasks (*MD* = −0.170, p_bonf_ = < 0.001), regardless of stimulus type.

### Response time: Effects of group, modality, and stimulus type

**Fig. 4:**
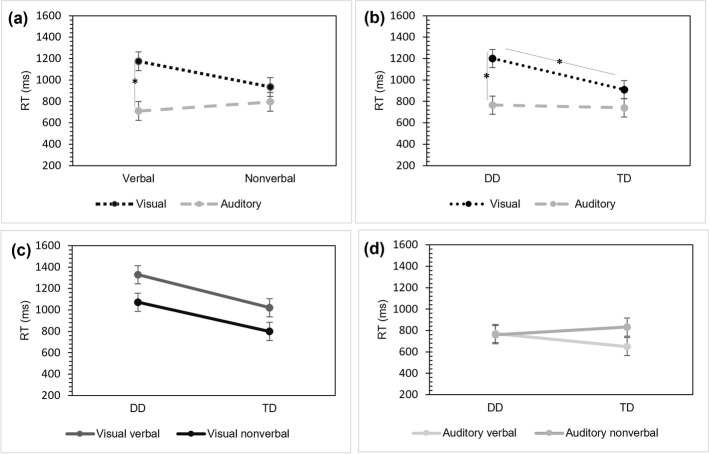
Response time for SL tasks. Note: RT in milliseconds for each modality (visual/auditory) by stimulus type (verbal/nonverbal) (**a**), for each modality broken by group (**b**), (**c**), and (**d**) for each SL task broken by group. Error bars display the standard error of the mean; *p < 0.05. The line (/) shows the interaction between variables (modality, stimulus type, and group). DD: dyslexia group, TD: Control group. In contrast to the interactions observed in the accuracy of the SL tasks, in the RT analysis, neither intragroup nor intergroup interactions were found involving stimulus type (see Fig. 4c and 4d)

We conducted a 2 × 2 × 2 mixed ANOVA on RT in SL tasks with Group (DD, TD) as the between-subjects factor, and the modality of task (visual, auditory) and stimulus type (verbal, nonverbal) as the within-subjects. We observed a main effect of Group, [*F* (1, 47) = 5.935, *p* = 0.019, ${\upeta }_{ p}^{2}$= 0.112], revealing that, overall, the DD group is slower during the forced-choice phase than the TD group. Regarding the within-subjects analyses, there were significant effects of Modality [*F*(1,47) = 25.661, *p* < 0.001, ${\upeta }_{ p}^{2}$= 0.353] indicating slower responses for the visual SL relative to the auditory SL task (*MD* = 301.827, p_bonf_ = < 0.001) and Modality by Stimulus type [*F*(1,47) = 6.308, *p* = 0.016, ${\upeta }_{ p}^{2}$ = 0.118], revealing that participants were slower during the verbal-visual SL task than in the auditory verbal task (*MD* = 464.042, p_bonf_ = < 0.001). but not with the nonverbal visual SL task relative to the nonverbal auditory tak (*MD* = 139.612, p_bonf_ = 0.693) (see Fig. 4a).

The Group by Modality [*F*(1,47) = 4.974, *p* = 0.031, ${\upeta }_{ p}^{2}$ = 0.09] interaction (see Fig. 4b), indicated that the DD group was slower in the visual modality as compared to TD (*MD* = 290.890, p_bonf_ = 0.008), but not when the modality is auditory (*MD* = 25.110, p_bonf_ = 1.000). This interaction also revealed that DD participants responded slower in the visual, relative to the auditory SL task (*MD* = 434.717, p_bonf_ = < 0.001). The remaining interactions involving the Group were not significant.

## Discussion

Understanding the underlying factors contributing to dyslexia, particularly from a neurocognitive perspective, remains a significant challenge. SL has emerged as a theoretical framework in research of neurodevelopmental disorders, enabling to examination of children's capacity to extract information concerning sequential patterns through mere exposure. On the subject of the acquisition of reading in transparent orthographies such as Spanish, the literacy acquisition process and access to written language require pattern extraction, comprehension of Spanish graphotactic relationships, and the formulation of hypotheses about the relationships between graphemes and phonemes in the language, so identifying the role of SL in children with dyslexia in transparent orthographies can help us better understand its relationship with the main literacy processes. The present study aimed to investigate the SL skills of Colombian children with dyslexia in comparison to their peers with typical reading abilities. Additionally, it sought to assess the impact of different modalities (auditory and visual) and stimulus type (verbal and nonverbal) on SL performance among participants who use a transparent orthography. Our data confirm our hypothesis that children with dyslexia in Spanish perform worse SL tasks than the control group, even in a transparent orthography that requires little implicit learning, and similarly to previous findings in deep orthographies (Kligler & Gabay, 2023).

Concerning accuracy in SL tasks, children with DD show significantly lower accuracy for all SL tasks independent of modality or stimulus type. These outcomes suggest that children with dyslexia in Spanish face challenges in extracting distributional probabilistic information when explicit instruction is lacking, and implicit learning skills are required to discern patterns, establish connections between information, and evaluate subsequent possibilities, which appear to be critical skills during literacy learning. Consistent with previous research (Schmalz et al., 2017), the recurring observation of this difference between the groups implies the existence of a deficient SL mechanism in children with dyslexia that may lead to poor learning of statistical assignments between sounds and letters (regularities of correspondence between graphemes and phonemes), as well as poor learning of visual representations of words and word fragments. (Frost et al., 2015). This highlights the possible importance of implicit learning skills for developing reading in Spanish, despite this language's transparent grapheme-phoneme relations.

By reviewing interactions in SL task accuracy by modality and by stimulus type, our findings identify that for the DD group performance was significantly lower for the visual modality tasks than for the auditory modality tasks, both in the intergroup and intragroup analyses. This result is related to that described by Sigurdardottir et al. (2017) who found that readers with dyslexia recognized on average fewer visual stimuli within the SL task than typical readers, suggesting that dyslexia may involve an impaired ability to pick up visual statistical regularities in the environment and that this impairment may be mediated by attention. Although in our experimental design, we did not directly assess attention, it was controlled by the coverage task in the familiarization phase. However, it would be interesting to extend the possible relationships between attention and SL in dyslexia, as recent studies show that there may be a relationship between different executive functions including attention and SL (Park et al., 2020).

Likewise, we observed that the challenge of achieving accuracy in the visual modality SL tasks intensifies within the DD group, particularly when the task involves verbal stimuli. In this case, we used pseudowords created from the AGL paradigm, which is an expected finding, since it is the SL task with the highest linguistic load and it is widely known that children with dyslexia present failures in the decoding of written words and pseudowords (Davies et al., 2007).

Although lower performance on auditory modality tasks has been observed in children with dyslexia due to auditory processing failures (Benson et al., 2020; Hämäläinen et al., 2013), other studies suggest a greater effect of SL on auditory tasks (Kahta & Schiff, 2019). However, research in SL indicates that auditory tasks may offer an advantage in resolution (Conway & Christiansen, 2005). Specifically, when stimuli are presented visually in a temporal stream, auditory learning appears superior to visual learning, highlighting differences in the acquisition of auditory and visual statistical patterns. Similarly, as described by Conway (2020), each subsystem (auditory, visual, motor) follows distinct trajectories in terms of cortical plasticity and maturation, and further research is required to explore the underlying mechanisms driving changes in statistical learning across different stages of development.

Along the same line, Qi et al. (2019) argue that assessing both modalities is crucial, as visual tasks may relate to the implicit acquisition of transition probabilities between letters, while auditory tasks may support the implicit acquisition of phonological skills. Thus, incorporating both modalities’ aids in clarifying the link that exists between orthography and phonology. Given that our study involves a transparent orthography, it is likely that children with dyslexia require less reliance on phonological skills, which are typically associated with auditory SL tasks. Consequently, they exhibit lower performance on tasks involving a higher grammatical complexity, such as visual verbal tasks. These findings align with previous research on the distinctive reading profiles of children with dyslexia in transparent orthographies (Davies et al., 2007; Jiménez et al., 2009).

Overall, the dyslexia group displayed longer reaction times on most SL tasks. However, when comparing the four tasks across different stimulus types between groups, the differences were not statistically significant. The cause of this slower response time remains unclear and, as Van der Kleij et al. (2018) noted, may be attributed to factors such as processing speed or delayed motor response in children with dyslexia. In this vein, previous research has indicated that children with dyslexia in transparent orthographies like German exhibit slow processing speeds in various sequential tasks involving visual item processing (Wimmer, 1993). In Finnish, Holopainen et al. (2001) highlighted that slow speed on rapid naming tasks is a significant predictor in dyslexia studies, especially in transparent languages even more so than measures of phonological awareness (Aguilar-Mediavilla et al., 2019).

When examining modality in response time for SL tasks, the DD group continued to be slower for visual tasks, but not for auditory tasks. Therefore, the DD group maintained the difficulty observed in accuracy, being the modality of the task a crucial aspect for the performance of the DD group (Singh et al., 2018). Additionally, the verbal stimulus tasks proved to be the most difficult ones. In this regard, Siegelman and Frost (2015) argue that SL is characterized not just by modality specificity but also by stimulus specificity and provide information that SL cannot be predicted as a uniform ability, but requires considering different related dimensions such as modality, stimulus characteristics and the characteristics of the subjects involved. In the same way, Frost et al. (2015) describe that SL evidences patterns of modality specificity and sometimes even stimulus specificity, emphasizing that each type of input (visual/auditory) has modality-specific constraints, which make for greater variability in the response to the SL task. This assumption goes in line with what has been reported by recent research (Hu et al., 2023; Kahta & Schiff, 2016) that relates that SL performance is mediated by mechanisms that are sensitive to the perceptual nature of the input across sensory modalities and in the case of our study, the DD group was sensitive to these modality and stimulus differences, being less accurate and taking longer in visual modality SL tasks.

Our results must consider some limitations, such as sample size. The exploratory nature of our research, particularly as it involves children with dyslexia who are speakers of a transparent orthography (Spanish), further emphasizes the need for careful consideration. Therefore, future studies might replicate this study with a larger sample of children in transparent orthographies using the four similar SL tasks analyzing two modalities and two stimulus type to demonstrate whether these results are consistent. Similarly, this research was supported by a control group matched by chronological age, but it would be interesting to analyze SL performance with groups matched by the reading level of the child with dyslexia, showing how SL behaves throughout the literacy learning process. Although the experimental tasks had a coverage task in the familiarization phase, it would be important in future research to measure visual and auditory attention as variables that may influence the response of children with dyslexia in SL tasks and allow to better isolate the effects of SL in this population.

In addition, our study evaluates the SL during the forced-choice phase, but not during the familiarization phase. This means that the study primarily captured the outcome of SL rather than the learning process itself. Future research would benefit from incorporating methodologies that measure SL during the stimulus exposure phase. Such an approach could provide deeper insights into the cognitive mechanisms at play during the learning process, thereby enhancing our understanding of how children with dyslexia engage with and process statistical regularities in real-time. Finally, our study examined intergroup performance in four SL tasks. In future studies, it would be interesting to investigate how performance on these SL tasks may eventually be related to achievement on reading, writing, and oral language tasks in Spanish for children with dyslexia.

## Conclusion

Our findings confirm that children with dyslexia learning to read in a transparent orthography like Spanish exhibit a deficit in statistical learning regardless of the modality or type of stimulus used. This suggests that children with dyslexia, whether in transparent or deep orthographies, struggle with extracting distributional probabilistic information without explicit learning instructions. These difficulties are particularly pronounced when the SL task involves visual modality and verbal stimuli. Although the dyslexia group generally responded slower across all tasks, it is accuracy, not response time, that is most significantly impaired in their SL tasks. Further research on the relationships between SL and transparent orthographies is crucial for a deeper understanding of this phenomenon.

## Supplementary Information

Below is the link to the electronic supplementary material.

Supplementary file1 (DOCX 37.8 KB)

## Data Availability

The data that support the findings of this study are available from the corresponding author upon reasonable request.

## Data accessibility

The data for this study are available in a repository hosted on the Open Science Framework (OSF) and can be accessed at the following link: [https://osf.io/4h5es/]. Researchers interested in utilizing the data may request access by contacting the study's investigators directly.
