# Supplementary material for: Impaired visual and verbal statistical learning in children with Dyslexia in a transparent orthography
Source: Ann Dyslexia. 2025 Jan 13;75(1):179–97. doi: 10.1007/s11881-024-00321-y (PMC11954696; doi:10.1007/s11881-024-00321-y)
Supplement: Supplementary file 1 — Supplementary file1 (DOCX 37.8 KB) [file 11881_2024_321_MOESM1_ESM.docx]

**Supplementary material**

**Table S1.** Descriptive statistics of the coverage task by SL task and by group.

| Modality | Stimulus type | Group | Mean | SD | SE | Coefficient of variation | Mean Rank | Sum Rank |
| --- | --- | --- | --- | --- | --- | --- | --- | --- |
| Visual | V | DD | 0.881 | 0.147 | 0.029 | 0.167 | 26.120 | 653.000 |
|  |  | TD | 0.887 | 0.124 | 0.025 | 0.140 | 24.880 | 622.000 |
|  | NV | DD | 0.548 | 0.065 | 0.013 | 0.119 | 19.000 | 475.000 |
|  |  | TD | 0.648 | 0.119 | 0.024 | 0.184 | 32.000 | 800.000 |
| Auditory | V | DD | 0.626 | 0.167 | 0.033 | 0.266 | 25.260 | 631.500 |
|  |  | TD | 0.632 | 0.164 | 0.033 | 0.260 | 25.740 | 643.500 |
|  | NV | DD | 0.640 | 0.112 | 0.022 | 0.175 | 24.700 | 617.500 |
|  |  | TD | 0.648 | 0.096 | 0.019 | 0.149 | 26.300 | 657.500 |

Note: *DD: Dyslexia Group; TD: Control Group; NV: Non-verbal; V: Verbal; SD: Standard Deviation; SE: Standard Error.*

**Table S2.** Mann-Whitney test of the coverage task by group: DD and TD

| SL Task | *U* | *p* | Rank-Biserial Correlation | SE Rank-Biserial Correlation |
| --- | --- | --- | --- | --- |
| Auditory verbal | 306.500 | 0.909 | -0.019 | 0.163 |
| Visual verbal | 328.000 | 0.765 | 0.050 | 0.163 |
| Visual Nonverbal | 150.000 | < .001* | -0.520 | 0.163 |
| Auditory Nonverbal | 292.500 | 0.684 | -0.064 | 0.163 |

Note: **p < .05*

**Table S3.** Spearman's Correlations Coverage task and accuracy SL tasks by DD group

| SL Task | *Spearman's rho* | *p* |
| --- | --- | --- |
| Auditory verbal | 0.125 | 0.550 |
| Visual verbal | 0.234 | 0.260 |
| Visual Nonverbal | -0.160 | 0.445 |
| Auditory Nonverbal | 0.071 | 0.736 |

Note: **p < .05*

**Table S4.** Spearman's Correlations Coverage task and accuracy SL tasks by TD group

| SL Task | *Spearman's rho* | *p* |
| --- | --- | --- |
| Auditory verbal | -0.145 | 0.488 |
| Visual verbal | -0.327 | 0.110 |
| Visual Nonverbal | 0.039 | 0.854 |
| Auditory Nonverbal | -0.048 | 0.821 |

Note: **p < .05*

**Table S5.** Mean of accuracy of the coverage task by participant

|  |  | Mean of accuracy | | | |
| --- | --- | --- | --- | --- | --- |
| Case | Group | Auditory Verbal | Visual Verbal | Visual Nonverbal | Auditory Nonverbal |
| 1 | DD | 0.50 | 0.75 | 0.50 | 0.50 |
| 2 | DD | 0.50 | 0.88 | 0.50 | 0.60 |
| 3 | DD | 0.75 | 1.00 | 0.50 | 0.80 |
| 4 | DD | 0.50 | 0.50 | 0.50 | 0.60 |
| 5 | DD | 0.88 | 1.00 | 0.50 | 0.60 |
| 6 | DD | 0.50 | 1.00 | 0.50 | 0.80 |
| 7 | DD | 0.75 | 0.88 | 0.60 | 0.70 |
| 8 | DD | 0.50 | 1.00 | 0.50 | 0.70 |
| 9 | DD | 0.88 | 0.94 | 0.50 | 0.50 |
| 10 | DD | 0.63 | 0.81 | 0.60 | 0.60 |
| 11 | DD | 0.50 | 0.81 | 0.60 | 0.60 |
| 12 | DD | 0.75 | 1.00 | 0.50 | 0.70 |
| 13 | DD | 0.63 | 1.00 | 0.60 | 0.70 |
| 14 | DD | 0.63 | 1.00 | 0.50 | 0.60 |
| 15 | DD | 0.50 | 1.00 | 0.60 | 0.50 |
| 16 | DD | 0.88 | 0.88 | 0.50 | 0.50 |
| 17 | DD | 0.88 | 1.00 | 0.50 | 0.90 |
| 18 | DD | 0.50 | 1.00 | 0.60 | 0.50 |
| 19 | DD | 0.50 | 0.81 | 0.70 | 0.60 |
| 20 | DD | 0.50 | 0.94 | 0.70 | 0.80 |
| 21 | DD | 0.50 | 0.50 | 0.50 | 0.60 |
| 22 | DD | 0.50 | 0.88 | 0.50 | 0.80 |
| 23 | DD | 0.50 | 0.69 | 0.60 | 0.60 |
| 24 | DD | 1.00 | 0.94 | 0.60 | 0.60 |
| 25 | DD | 0.50 | 0.81 | 0.50 | 0.60 |
| 26 | TD | 0.88 | 0.94 | 0.90 | 0.80 |
| 27 | TD | 0.50 | 1.00 | 0.60 | 0.60 |
| 28 | TD | 0.75 | 0.94 | 0.60 | 0.70 |
| 29 | TD | 0.50 | 1.00 | 0.80 | 0.80 |
| 30 | TD | 0.75 | 0.94 | 0.50 | 0.90 |
| 31 | TD | 0.50 | 1.00 | 0.50 | 0.60 |
| 32 | TD | 0.88 | 0.88 | 0.80 | 0.70 |
| 33 | TD | 0.50 | 0.88 | 0.60 | 0.60 |
| 34 | TD | 0.75 | 0.94 | 0.60 | 0.60 |
| 35 | TD | 0.50 | 0.88 | 0.70 | 0.60 |
| 36 | TD | 0.50 | 0.94 | 0.50 | 0.60 |
| 37 | TD | 0.50 | 1.00 | 0.50 | 0.60 |
| 38 | TD | 0.50 | 0.69 | 0.60 | 0.60 |
| 39 | TD | 0.75 | 0.94 | 0.60 | 0.70 |
| 40 | TD | 0.38 | 0.44 | 0.60 | 0.60 |
| 41 | TD | 0.75 | 0.75 | 0.60 | 0.60 |
| 42 | TD | 0.50 | 0.88 | 0.60 | 0.60 |
| 43 | TD | 0.88 | 1.00 | 0.60 | 0.60 |
| 44 | TD | 0.75 | 0.88 | 0.60 | 0.70 |
| 45 | TD | 0.50 | 0.88 | 0.80 | 0.50 |
| 46 | TD | 0.38 | 0.94 | 0.60 | 0.50 |
| 47 | TD | 0.63 | 0.81 | 0.70 | 0.70 |
| 48 | TD | 0.88 | 0.75 | 0.80 | 0.80 |
| 49 | TD | 0.63 | 0.94 | 0.60 | 0.60 |
| 50 | TD | 0.75 | 0.94 | 0.90 | 0.60 |

Note: *DD: Dyslexia Group; TD: Control Group*
